# Rapid and Sensitive Detection of Cardiac Troponin I for Point-of-Care Tests Based on Red Fluorescent Microspheres

**DOI:** 10.3390/molecules23051102

**Published:** 2018-05-07

**Authors:** Yanxue Cai, Keren Kang, Qianru Li, Yu Wang, Xiaowei He

**Affiliations:** 1 School of Food Science and Engineering, South China University of Technology, Guangzhou 510640, China; yanxue.cai@foxmail.com (Y.C.); qianruli@163.com (Q.L.); wyhgn3344@163.com (Y.W.); 2 National & Local United Engineering Lab of Rapid Diagnostic Test, Guangzhou Wondfo Biotech Co., Ltd., Guangzhou 5l0663, China; keren.kang@protonmail.com; 3 Fisheries College, Guangdong Ocean University, Zhanjiang 524088, China

**Keywords:** cardiac troponin I, core-shell microspheres, lateral flow immunoassay, point-of-care tests, clinical application

## Abstract

A reliable lateral flow immunoassay (LFIA) based on a facile one-step synthesis of single microspheres in combining with immunochromatography technique was developed to establish a new point-of-care test (POCT) for the rapid and early detection of cardiac troponin I (cTnI), a kind of cardiac specific biomarker for acute myocardial infarction (AMI). The double layered microspheres with clear core-shell structures were produced using soap-free emulsion polymerization method with inexpensive compounds (styrene and acrylic acid). The synthetic process was simple, rapid and easy to control due to one-step synthesis without any complicated procedures. The microspheres are nanostructure with high surface area, which have numerous carboxyl groups on the out layer, resulting in high-efficiency coupling between the carrier and antibody via amide bond. Meanwhile, the red fluorescent dye, Nile-red (NR), was wrapped inside the microspheres to improve its stability, as well to reduce the background noise, because of its higher emission wavelength than interference from real plasma samples. The core-shell structures provided different functional areas to separate antibody and dyes, so the immunoassay has highly sensitive, wide working curves in the range of 0–40 ng/mL, low limits of detection (LOD) at 0.016 ng/mL, and limits of quantification (LOQ) at 0.087 ng/mL with coefficient of variations (CV) of 10%. This strategy suggested an outstanding platform for LFIA, with good reproducibility and stability to straightforwardly analyze the plasma samples without washing steps, thereby reducing the operating procedures for non-professionals and promoting detection efficiency. The whole detection process can be completed in less than 15 min. This novel immunoassay offers a reliable and favorable analytical result by detecting the real samples, indicating that it holds great potential as a new alternative for biomolecule detection in complex samples, for the early detection of cardiac specific biomarkers.

## 1. Introduction

Cardiac troponin I (cTnI) is a cardiac specific biomarker that is released during myocardial necrosis; it reaches a peak value in blood after about 11 h [1]. Compared to myoglobin and creatine-kinase-MB, cTnI is more sensitive and specific to acute myocardial infarction (AMI) [2]. As such, it has been recommended as a fundamental cardiac marker for the diagnosis of AMI [3]. Therefore, developing a convenient assay for cTnI detection, in line with rapid and accurate early detection requirements, is very useful for heart-disease prevention.

To date, various analysis techniques have been developed for cardiac troponin detection, including the use of chemiluminescent immunoassays [4], enzyme-linked immunosorbent assays [5], electrical detections, and the use of aptamer-based biosensors [6,7]. In order to improve detection sensitivity and the limit of detection (LOD), researches were mainly focused on new strategies such as pulse laser technology [8], upconversion rare-earth probes, and surface plasmon resonance (SPR) technology [9,10]. Although those techniques are expected to pave the way for improved specificity and sensitivity, the demand for extremely expensive devices and cumbersome operation both greatly limits their feasibility. Point-of-care tests (POCT) based on enzyme-linked immunosorbent assays (ELISA), fluorescence and chemiluminescent have been developed for use in the rapid detection of AMI. Lateral flow immunoassay (LFIA) based test strip is one of the key technologies in the POCT, due to its simplicity, rapidity, and ease-of-use.

During the detection process of LFIA, contrast to foodborne pathogens and environmental pollutants, cTnI is affected by the autofluorescence of interfering factors from blood samples, resulting in low analytical sensitivity and false positive results [11]. Therefore, a series of good performance probes have been developed to improve the sensitivity, including quantum dots, carbon dots, up conversion rare-earth materials, and organic dyes such as fluorescein isothiocyanate (FITC) and Rubpy [12,13]. Some of the probes display the high sensitivity and specificity required for the immunoassay. However, their low stability and reproducibility are still bottlenecks to their practical application. For the immunoassay test strip technique, it is important to improve the sensitivity and LOD for the detection target, and to maintain excellent stability and repeatability of the assay to meet the requirements of practical and commercial applications. The organic dye molecules are usually exposed to severe environmental conditions during use and storage, and often suffer from photobleaching and quenching due to the influences of solvent molecules and reactive species, such as oxygen or oxidative ions in the solution [14]. Accordingly, the design of dye-doped nanoparticles, instead of dye molecules, for diagnostic has received more attention in recent years. Compared to dye molecules, dye-labeling nanoparticles exhibit significantly enhanced brightness and stability, and could improve reproducibility in practical use.

Generally, polymers as common materials have obvious advantages in the preparation of nanoparticles, in controlling functional groups and structure. However, in order to conjugate the antibody proteins, the synthesis usually requires at least two steps, including the establishment of the principal part and the modification of nanoparticles surface. In previous studies, a core-shell microsphere was synthesized using polystyrene as the core at first; the shell was then built using acrylamide as a template to capture adenosine, which was used as a bridge for conjugating antibody proteins [15]. Protein immobilization onto the microspheres can also be carried out through physical adsorption and electrostatic interactions, which was based on controlling of the surface charges by changing the percentage of styrene and covering the microspheres surface with bacterial outer membrane to increase the affinity of antibodies [16]. In addition, inorganic and metal materials were also considered as alternative choices due to their stable morphologies and special properties [17]. Among them, silica microspheres were usually used as a carrier with functionalized surface modification, such as nano-gold and graphene oxide [18], for enhancing the signal. Fe_3_O_4_ [19] and Co_3_O_4_ [20] were also frequently applied to impart the functionality of magnetic enrichment and high catalytic activity. In order to conjugate the antibodies for immunoassay, some functional groups, such as carboxyl and amidogen, still need to be modified on those microsphere surface. Although these methods are effective in improving the detection sensitivity and product stability, many problems are still encountered, such as complicated synthetic steps, low yield, and high cost, which limit their application in clinical detection.

In this study, a facile one-step synthesis was used to prepare a single microsphere with clear core-shell structures to establish a new POCT method for the rapid and early detection of cTnI. This detection method, based on double layered fluorescent microsphere in combination with immunochromatography technique, was more challenging than previous works, since it needed to have accurate test results as well as rapidity and convenience in practical application. The microsphere was produced using relatively inexpensive compounds (styrene and acrylic acid) to improve the stability of the red fluorescent dye Nile-red (NR), which could reduce the background noise interference signal. Furthermore, this assay can be used in straightforward analysis of the plasma samples without washing steps, thereby reducing the operating procedures for non-professionals and improving detection efficiency. Our results indicate that this assay could be a used for the early detection of cTnI in the future.

## 2. Results and Discussion

### 2.1. One-Step Synthesis of Microspheres and Dye-Doped Procedure

**Figure 1:**
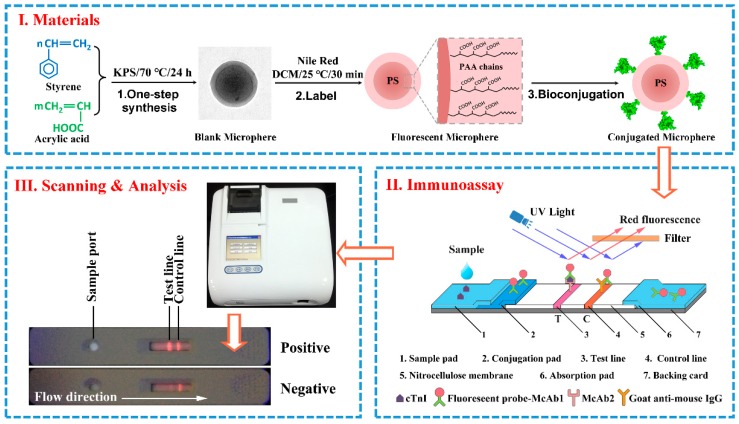
The schematic representation of (**I**) The synthesis of fluorescent immune-microspheres; (**II**) Immunoassay mechanism lateral flow test strip and (**III**) Scanning & analysis process.

Functional nano-spheres with different reactive groups on the surface are popular in various fields, such as chromatography and biomedical analysis [21,22]. The carboxyl group is one of the most important functional groups for biomedical applications. In our procedure, a facile one-step polymerization was used to prepare the core-shell microspheres. The synthetic process illustration was shown in Figure 1-I. Acrylic acid was applied to synthesize the surface carboxyl functionality of the polystyrene particles through copolymerization with styrene that was initiated with potassium peroxodisulfate (KPS). This one-step synthesis has significant advantages compared to conventional emulsion polymerization, which has two or more stages [23,24]: preparation of the main polystyrene core, and modifying the surface with functional groups. In this polymerization process, the hydrophilic difference of the comonomer that absorbed by polymer contributes to the clear core-shell structure which hydrophobic polystyrene as the core and hydrophilic poly-acrylic acid as the shell around the particle. It is simple and easy to operate, without using any high-temperature and high-pressure equipment. Furthermore, this synthetic method, without any additional surfactants, could improve the colloidal stability of the microspheres in the aqueous phase, and conjugate foreign molecules. The functional groups were laid on the outer layers of the particles and used for the subsequent incorporation of anti-cTnI antibody.

Fluorescent dye-doped polymer particles have been used in many applications, such as biosensors and tumor imaging [25]. The emission of the fluorescent probe in common LFIA is about 475 nm, which is usually obscured by the background, due to the autofluorescence from the sample matrix. Compared to foodborne pathogens and environmental pollutants, a stronger interferential autofluorescence from some biomolecules in plasma, such as pyridoxine, bilirubin, collagen and NAD(P)H, is observed in the range of 300–450 nm [26]. These interferential autofluorescence may result in low analytical sensitivity in the immunoassay. In contrast, NR could be used as an alternative to distinguish the emission wavelength from the autofluorescence of plasma samples. As shown in Figure S1, the maximum absorption of NR loaded microspheres was 542 nm, and the emission wavelength was 584 nm (excitation: 544 nm), which effectively avoids the major absorption and autofluorescence wavebands from plasma samples, and reduces the impact of background noise.

**Figure 2:**
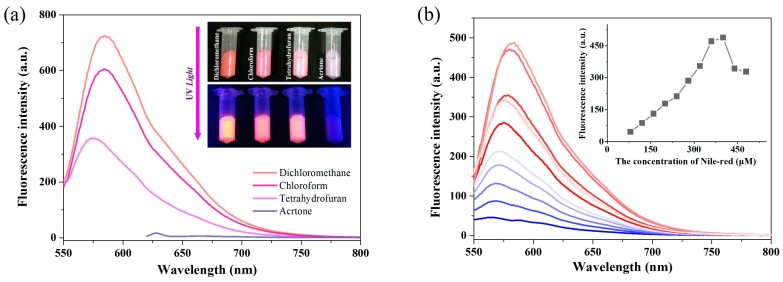
(**a**) The spectrum and photo of microspheres with different solvents; (**b**) The fluorescence intensity at different concentration of NR with the DCM-water as solvent.

A facile, flexible and reproducible procedure is of considerable importance for the preparation of fluorescent particles. The straightforward swelling procedure was applied for loading fluorophores to the carboxyl-functionalized polystyrene microspheres, which helped to retain the native surface groups of microspheres at the same time. In order to optimize the swelling procedure, THF, acetone, chloroform and DCM were investigated as swelling solvents. As shown in Figure 2a, at the same conditions, the highest fluorescent intensity was obtained using DCM-water system as a solvent. A bright red fluorescence was displayed in the dye-embedded particles under a UV-light (Figure 2a, insert). In addition, the loading efficiency of NR into the microspheres could be easily controlled by applying different concentrations of the dye solution. Figure 2b shows that concentrations higher than 400 μM could lead to fluorescence self-quenching, so the optimum concentration of NR was determined to be 400 μM for the following application.

### 2.2. Characterization of Microspheres

**Figure 3:**
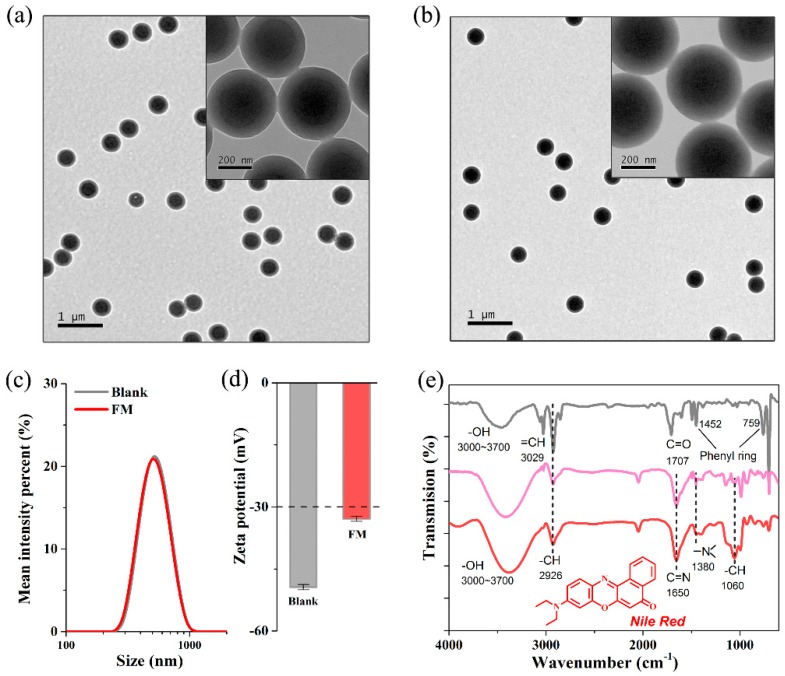
The TEM images of (**a**) blank microspheres and (**b**) fluorescent microspheres; (**c**) The particle size and (**d**) ζ-potential of blank and fluorescent microspheres (FM); (**e**) The FTIR spectrum of blank microspheres (grey line), NR (pink line) and fluorescent microspheres (red line).

The TEM images of the blank and fluorescent microspheres are given in Figure 3a,b, which clearly show a discernable core-shell structure with an average size of ~500 nm. The enlarged image (inset of Figure 3a,b) suggests that the thickness of the polyacrylic acid shell layer was ~80 nm. In addition, the blank microspheres had a clearer boundary than the dyed microspheres, which may be due to swelling in the dyeing process. The particle size distribution (Figure 3c) confirmed the TEM characterization. The Z-average partial sizes of blank and fluorescent microspheres were 532.2 ± 17.9 nm and 543.7 ± 14.5 nm respectively, implying that the microsphere sizes were not significantly changed during the dyeing process. Furthermore, the ζ-potential of the blank and fluorescent microspheres were −49.4 ± 0.6 and −32.9 ± 0.59 mV (Figure 3d). Their absolute values (higher than 30 mV) reveal that they both had excellent dispersion and stability, due to high electrostatic repulsion among the nanoparticles [27]. A uniform and stable structure is the basis for the use of the microspheres for creating a rapid detection method.

The FTIR spectra of blank and fluorescent microspheres samples are shown in Figure 3e. In the two spectra, the broad absorption band from 3000 to 3700 cm^−1^ represents the -OH stretching vibration [28], which was regarded as a result of the presence of water due to the hydrophilic carboxyl groups on the surface of microspheres. For blank microspheres, peaks at 1707 cm^−1^ were assigned to the C=O stretching from the acrylic acid [29], and peaks at 1452 cm^−1^ and 759 cm^−1^ were attributed to the characteristic peaks of phenyl ring from styrene [30], which were consistent with the chemical structure of synthetic materials. After the dyeing process, some new characteristic peaks appeared from NR, including the peaks at 1650 cm^−1^, 1380 cm^−1^ and 1060 cm^−1^, which belong to C=N stretching vibration, nitrogen heterocycle and -CH bending in the aromatic heterocycle [31], indicating that the NR have been loaded into the blank microspheres.

### 2.3. Conditions Optimization and Immunoassay Procedures

In order to obtain the best performance, the reaction time and antibody concentration for coupling between the antibody and the microspheres were optimized. As shown in Figure S2a, the best response value was obtained when the reaction time was 120 min. Antibody concentration was also an important parameter for improving efficiency and controlling costs. As shown in Figure S2b, 500 μg/mL was effective for coupling. Therefore, 500 μg/mL was considered to be the optimum amount and was used in subsequent experiments. Furthermore, the immunoreaction time of the strip test is generally believed to be the most significant factor influencing fluorescence intensity during the assay. As shown in Figure S3a, the ratio of the fluorescence peak heights of T-line and C-line (H_T_/H_C_) was used to evaluate the effect of immunoreaction time between the antibody and antigen over a range of 3–33 min. As can be seen, the ratio of H_T_/H_C_ significantly increased up to 9 min, and reached an approximately stable value after 15 min, indicating that this was a suitable incubation time.

Figure 1-II illustrates the principle of the LFIA for measuring cTnI, based on the sandwich-type method. Briefly, the steps taking place in the strips were as follows: firstly, each sample (75 μL) containing cTnI was added onto the sample pad. Subsequently, the sample migrated toward the particles labeled cTnI McAb1 on the conjugate pad, and then the complexes were captured by cTnI McAb2 that was coated on the NC membrane as the T-line. Next, the C-line captured the excess microspheres. Lastly, the surplus nanoparticle complexes migrated into the absorption pad by capillary action. After the immunoreaction process, the fluorescence intensity was estimated from fluorescent microspheres on the T-line and C-line, and the results were quantified by the laboratory made Feice Lateral Flow Reader (Wondfo Biotech Co., Ltd., Guangzhou, China) as shown in Figure 1-III.

Once a patient has the clinical features of AMI, disease development is very fast, and early diagnosis and treatment are very important for patient recovery. In general, diagnostic strategies with electrocardiograph have been used to rule out acute AMI in 6 to 12 h. The diagnostic time is reduced to 90 min [32], or even about 15 min [33], with the development of POCT technology. In this immunoassay, the whole detection process can be completed less than 15 min to get the final testing results. Moreover, the test strip remained stable after 3 months storage at 4 °C. Due to these advantages, the test is easy to popularize and apply.

### 2.4. Performance Analysis of Immunoassay

**Figure 4:**
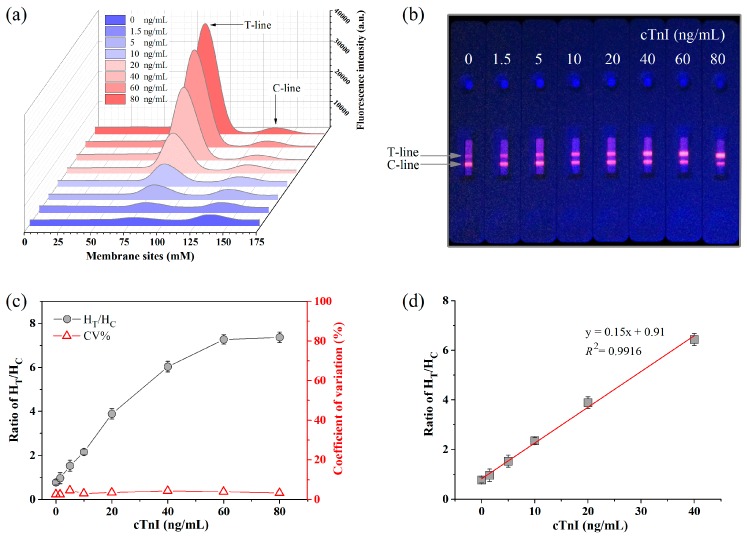
(**a**) The fluorescence signal intensity at different sites on the NC membrane at cTnI concentration from 0 to 80 ng/mL; (**b**) The photo of detection strips under UV-light; (**c**) The ratio of H_T_/H_C_ and the corresponding coefficient of variations; (**d**) The standard curve of cTnI test.

Under optimized immunoreaction conditions, the standard curve was constructed to measure the analytical performances of the proposed NR doped particles-based strip. As shown in Figure 4a, the strips showed different fluorescence signal intensities at different sites on the NC membranes. The most intense signal was on the T-line zone. The corresponding results can also be visually observed from the photos of those strips under a UV-light (Figure 4b). Based on the ratio of H_T_/H_C_, the relative fluorescence intensity rose with increasing concentration of cTnI, and no high-dose hook effect was observed when the concentration reached 80 ng/mL, as shown in Figure 4c. In addition, the CV of the fluorescence intensity between replicates (*n* = 5) was in the range of 2.58–4.6% at different concentration of cTnI, indicating that the result was reliable and had excellent stability. The detection concentration of cTnI was found to be linear in the range of 0~40 ng/mL, as shown in Figure 4d. Generally, a concentration of cTnI < 0.3 ng/mL is considered to be normal and safe; abnormal levels are from 0.3 to 40 ng ng/mL and higher [34]. Thus, the linear range of this immunoassay conforms to the early diagnostic conditions for AMI.

**Figure 5:**
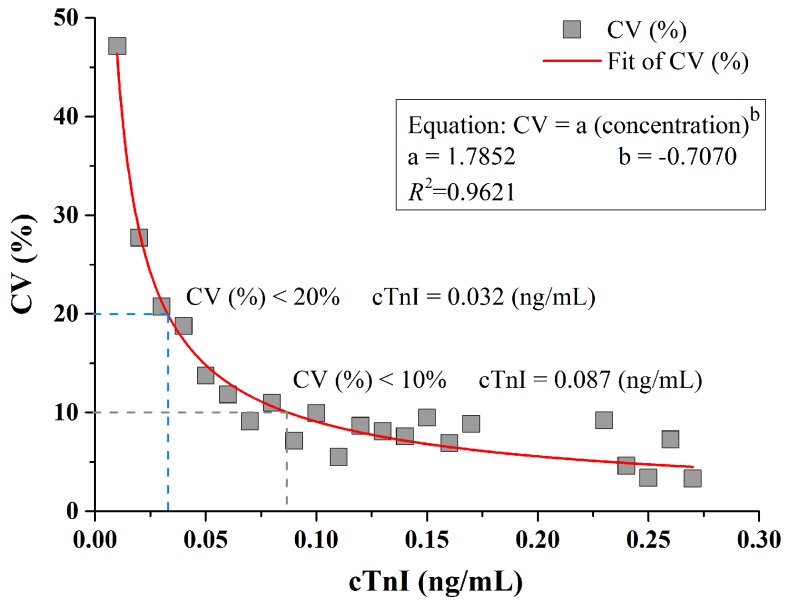
The cTnI concentration of LOQ at 10% CV and 20% CV from 0 to 0.30 ng/mL.

As a standard biomarker for the diagnosis of heart attacks, high sensitivity is required for cTnI assay. In this work, the limit of blank (LOB) and LOD were calculated based on the Clinical Laboratory Standards Institute (CLSI) Guideline EP17-A2 [35]. LOB was determined as 0.013 ng/mL (mean of zero calibrator + 1.645 times the standard deviation, *n* = 20), and LOD was determined to be 0.016 ng/mL (3 times the standard deviation of the blank, *n* = 20). Table 1 lists some previous cardiac troponin assays for the LOD values of cTnI detection. Although the present method was not at the lowest level compared to some other methods with large instruments in the lab, the proposed immunoassay also achieved excellent analysis performance, meeting the LOD requirements of cTnI POCT detection. Furthermore, the limit of quantification (LOQ) was calculated at the lowest cTnI concentration measured, with CV of 10% and 20%, to evaluate the effectiveness of this method (Figure 5). The LOQ values were 0.087 ng/mL with CV of 10%, and 0.032 ng/mL with CV of 20%, in comparing with the previous reports and some commercial manufacture in Table 2, which also showed a good performance to early detection of cTnI.

The reproducibility, stability and specificity of an immunoassay are important factors for practical applications. Intra-assay measurement precision was used to evaluate the reproducibility of cTnI detection with three concentration levels (2.40, 19.83 and 40.42 ng/mL) for ten test strips in the same batch. For the inter-assay precision, each concentration of cTnI was measured by nine test strips, which were randomly chosen from three different batches. The results are shown in Table 3. The calculated intra-assay and inter-assay CV were lower than 5% at cTnI concentration of 2.40 and 19.83 ng/mL, and the CV were lower than 10% at 40.42 ng/mL cTnI. The results indicate that the immunoassay displayed a good level of precision, and is thus suitable for cTnI strip quantitation in on-site tests.

In practical applications, the stability of the test method is a very important parameter for performance analysis, but has been largely unreported previous literature. In this work, the stability of the strip was studied through storage and accelerated test, as shown in Table S1. The effectiveness of the test strips was maintained in storage at 4 °C for three months, and under accelerated tests at 50 °C for 28 days. The results displayed acceptable stability in the proposed immunoassay during commercial storage and usage. In addition, the specificity was determined by evaluating its reactivity with some interfering factors, including bilirubin, cholesterol, sodium aside and hemoglobin, which are the major interference factors in real plasma samples. As shown in Table 4, two levels of concentrations of cTnI were used for the assay, in order to obtain reliable results. The results showed that the relative deviations (RD) were all in the range of ±10%, suggesting that the effects of interfering factors were not significant, and the specificity of this immunoassay was acceptable as its highly specific toward cTnI.

### 2.5. Clinical Test of cTnI with Plasma Samples

**Figure 6:**
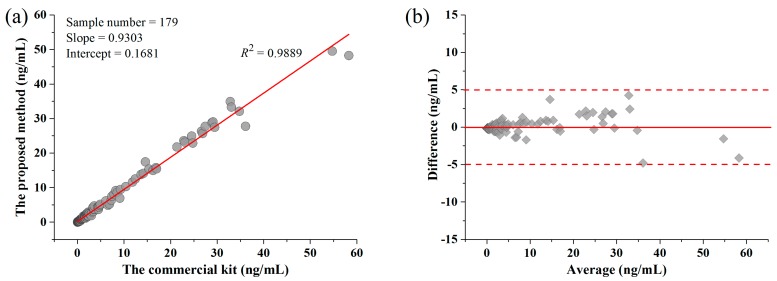
The results clinical test of cTnI (**a**) and the average difference (**b**).

In order to evaluate the practical application of the double layered microspheres-based method for the cTnI assay, a total of 179 plasma samples which were supplied by Guangzhou Wondfo Biotech Co., Ltd. (Guangzhou, China), including 56 low level concentration samples (<0.3 ng/mL), 86 median value samples (0.3–5 ng/mL) and 37 high value samples (>5 ng/mL), were analyzed by the developed test strips. The results were compared to the results from a chemiluminescence assay. Comparisons of both plasma results were made based on the Bland-Altman plot, and a Passing-Bablok regression analysis, as shown in Figure 6. The correlation coefficient (*R*^2^) for the regression line of the Passing-Bablok regression analysis was 0.9889, indicating a good linear relationship between the two measurements. The mean relative difference (95% limits of agreement) was 0.2 from Bland-Altman plot results, revealing that there was no significant bias between these two methods. It can be concluded that the immunoassay demonstrates good accuracy in clinical testing. This immunoassay could be used as the on-site assay for patients, within the capacity of rapid and accurate quantification of the cTnI, which is very important for early screening of patients with AMIs, as well as for the diagnosis and treatment of the disease.

## 3. Materials and Methods

### 3.1. Reagents

Styrene (98%; Aladdin, Shanghai, China) was washed with a 2 wt % NaOH aqueous solution, as well as Milli-Q water, followed by drying with CaCl_2_, (Tianjin Jinke Fine Chemical Plant, Tianjin, China), and distilling under reduced pressure. Other reagents and solvents were commercial products and were used without further purification: KPS, acrylic acid, Nile-red, tetrahydrofuran (THF), chloroform, dichloromethane (DCM) and acetone were all analytical grade and purchased from Aladdin Reagent Co. (Shanghai, China). 1-ethyl-3-(3-dimethylaminopropyl)-carbodiimide (EDC), *N*-hydroxysuccinimide (NHS), bovine serum albumin (BSA) and 2-(*N*-morpholine)-ethane sulphonic acid (MES, ultra-pure grade, 99.0%) were purchased from Sigma-Aldrich Co. (St. Louis, MO, USA). The antibodies were created from the HyTest Ltd. (Turku, Finland). A monoclonal antibody (mAb) recognizing the epitope at amino acids (AA) 41–49 (19C7-mAb) was used as a detection antibody. Two antibodies, a mAb recognizing the epitope at AA 18–28 (M18-mAb), and a mAb recognizing the epitope at AA 82–93 (560-mAb), were used as capture antibodies. The water used during the experiments was purified through a Milli-Q purification system.

### 3.2. One-Step Soap Free Emulsion Polymerization Procedure

Carboxyl-functionalized polystyrene microspheres were prepared by soap-free emulsion polymerization using KPS as the initiator. The experimental conditions have been optimized repeatedly and the following procedure was used: briefly, 0.1 g KPS was dissolved in 100 mL water mixed with 7.0 g styrene and 0.5 g acrylic acid, the mixer was added into a 250 mL three-neck reaction flask equipped with a condenser under nitrogen atmosphere, and then heated to 70 °C for 24 h under stirring. The emulsion particles were collected by centrifugation, and cleaned by repeated centrifugation and ultrasonic dispersion in water.

### 3.3. Preparation of Fluorescent Nanoparticles via Swelling

The fluorescent dye was inserted into the microspheres by swelling. A 5.0 mL aqueous suspension of emulsion particles (2 wt %) was put into 15.0 mL centrifuge tube, to which 4.0 mL Milli-Q water and 1.0 mL 2% sodium dodecyl sulfonate were added. The NR, at a concentration of 0.8 mg/mL, was added to the emulsion particles suspension with different swelling solvents, including THF, acetone, chloroform and DCM, which were optimized to obtain the best experimental conditions. The mixture was dispersed by a dispersion homogenizer (Fluko, Shanghai, China) at medium speed at 25 °C for 30 min, and then kept open to the atmosphere at 40 °C for 4 h to remove the organic solvents. The unloaded NR in solution was removed by washing the emulsion particles as follows: the prepared dye-doped nanoparticles were first centrifuged at 12,000× *g* for 20 min at 10 °C, and the precipitates were collected. The collected precipitates were then ultrasonically dispersed in water under centrifugation (12,000× *g*, 20 min, and 10 °C), to remove the supernatant and dispersed in Milli-Q water. The dye-doped nanoparticles were triple-rinsed with Milli-Q water, and finally dispersed in water and stored at 4 °C.

### 3.4. Preparation of Bioconjuated Microspheres

Using EDC/NHS as a coupling agent, the anti-cTnI was covalently immobilized onto microspheres via amide bond formation between carboxyl of carboxyl-functionalized microspheres and the amine groups of the anti-cTnI antibody [44]. Briefly, the microspheres were added to a 10 mM MES buffer (pH 7.0) with 10 mM EDC and 10 mM NHS to activate the carboxylic groups, for 15 min in dark. After a buffer exchange using a 10 mM MES buffer (pH 8.0), 0.4 g/L anti-cTnI McAb1 was added to the activated microspheres. After a 2 h coupling reaction at room temperature, the uncoupled antibody was removed by centrifugation at 12,000× *g* for 10 min at 4 °C. Then the remaining active groups on microspheres surface were blocked with 50 mM glycine (0.1% Tween-20) for 1 h. Finally, the conjugates were stored in 10 mM Tris-buffer (pH 8.5) with 0.5 g/L NaN_3_ and 0.1% Tween-20.

### 3.5. Fabrication of the Lateral Flow Test Strip

The fluorescent microsphere-based lateral flow test strip consisted of five parts, as follows: the sample application pad, conjugate pad, nitrocellulose (NC) membrane, absorption pad, and a backing plate, as shown in Figure 1-II. The sample pad (20 mm × 30 cm) and the conjugate pad (13 mm × 30 cm) were both made of glass fiber. The sample application pad was first blocked with a blocking buffer (0.01 M PBS (pH 7.4) containing 2% PEG 6000, 1% BSA, and 2% Tween-20), followed by overnight drying at 40 °C. The conjugate pad was prepared by dispensing the desired volume of fluorescent microsphere-coupled McAb1 onto the glass fiber through a HGS510 dispenser (Hangzhou Autokun Technology Co., Ltd., Hangzhou, China), followed by drying at 40 °C for 12 h. To prepare the NC membrane, the anti-cTnI McAb2 and goat anti-mouse IgG were separately spotted onto the NC membrane with an IsoFlow dispenser (Imagene Technology, Lebanon, NH, USA) at a jetting rate of 0.5 μL/cm to generate test line (T-line) and control line (C-line), leaving 0.5 cm between the two lines, followed by drying at 50 °C for 48 h. The absorption pad was used without any treatment. Finally, the sample pad, conjugate pad, NC membrane, and absorption pad were laminated onto a backing plate and then cut into 4 mm wide strips using a HGS201 cutter (Hangzhou Autokun Technology Co., Ltd., Hangzhou, China), which were assembled into strip cassettes for the following assay.

### 3.6. Characterization

The morphologies of the microspheres were characterized using a transmission electron microscope (TEM) (HT7700, Hitachi, Japan). Fluorescence measurements were recorded on a fluorescence spectrophotometer (RF-5301PC, Shimadzu, Japan). The particle size distribution and ζ-potential of blank microspheres and fluorescent nanoparticle were determined using a laser particle size analyzer (Zeta Sizer Nano-S90, Malvern, UK). Fourier transform infrared spectroscopy (FTIR) was used to determine the changes in functional groups, using a FTIR spectrometer (Equinox 55 Bruker Banner Lane, Coventry, Germany). More experimental details are given in the supplementary text.

### 3.7. Statistical Analysis

Statistical analysis on a completely randomized design was conducted using the one-way analysis of variance (ANOVA) procedure, with SPSS 17.0 software, at a level of significance set at *p* = 0.05. Bland-Altman plot and Passing-Bablok regression analyses were performed with MedCalc Software (MedCalc, Mariakerke, Belgium). All data were presented as mean values with their standard deviations (mean ± S.D.). Differences were accepted as significant when *p* < 0.05.

## 4. Conclusions

In conclusion, based on a simple synthesis of red fluorescent nano-microspheres in combination with the lateral flow immune technique, a rapid, accurate, and stable assay was established for the on-site quantitative detection of cTnI. The design of the double layered microspheres uses core-shell structures to divide functional areas for separating antibody and probes, avoiding the antibody inactivation and dye fluorescence quenching caused by their interactions. The structure enabled the capture of the antibody onto the microsphere outer layer, to improve the stability of test strips. Owing to the red fluorescence inside the microspheres with a high wavelength which helps to avoid the background noise from samples of plasma, the proposed method displayed high sensitivity, wide range, and low LOD. These features, together with other advantages such as user friendliness and convenient operation, showed this platform to be a reliable and favorable assay for real samples. This new approac, holds great potential as an alternative for the detection of biomolecules in complex samples for the early diagnosis of the cardiac specific biomarker cTnI.

## Figures and Tables

**Table 1:** The comparison of performance for cTnI from reported literatures and current work.

Method	LOD	Stability	Time
Cationic isotachophoresis [36]	46 ng/mL	-	~10 min
Wavelength modulation SPR biosensor [10]	37.5 ng/mL	-	~70 min
Fluorogenic noncompetitive immunoassay [37]	6.7 ng/mL	-	~19 min
Capillary tube indicators [38]	0.1 ng/mL	-	~20 min
Electrochemical immunosensor [39]	0.05 ng/mL	30 days	~15 min
Red florescent microspheres immunoassay (This work)	0.016 ng/mL	90 days	~15 min
Pt NPs modified hybrid film immunosensor [40]	1 pg/mL	1 month	~20 min

**Table 2:** The comparison of limit of quantification (LOQ) for cTnI from reported literatures and current work.

Method	LOQ (ng/mL)	
	CV < 10%	CV < 20%
Immunoassay sandwich fluorescence ( bioMerieux VIDAS) [41]	0.11	-
Enzyme-linked immunosorbent assay (i-STAT) [41]	0.10	0.07
Microfluidic immunochip assay [42]	-	0.042
Homogeneous sandwich immunoassay [43]	-	0.038
Red florescent microspheres immunoassay (This work)	0.087	0.032

**Table 3:** Results of reproducibility analysis by intra-assay and inter-assay precision.

cTnI (ng/mL)	Intra-Assay Precision (*n* = 10)		Inter-Assay Precision (*n* = 9)	
	Mean ± SD (ng/mL)	CV (%)	Mean ± SD (ng/mL)	CV (%)
2.40	2.32 ± 0.09	3.86	2.54 ± 0.12	4.69
19.83	19.28 ± 0.51	2.67	20.40 ± 0.87	4.26
40.42	41.58 ± 2.57	6.18	39.47 ± 2.80	7.10

**Table 4:** The specificity study of the developed method with different interferons.

Interfering Substance	cTnI (1.3 ng/mL)		cTnI (2.85 ng/mL)	
	Value	RD (%)	Value	RD (%)
Control	1.31 ± 0.01	0.77	2.93 ± 0.08	2.81
Bilirubin (2 ng/mL)	1.24 ± 0.02	−4.62	2.69 ± 0.09	−5.61
Cholesterol (15 ng/mL)	1.31 ± 0.04	0.77	2.64 ± 0.01	−7.37
Sodium aside (6 ng/mL)	1.27 ± 0.01	−2.31	2.76 ± 0.05	−3.16
Hemoglobin (10 ng/mL)	1.23 ± 0.05	−5.38	2.75 ± 0.07	−3.51

Note: RD = (Value − Standard value)/Standard value.

## Supplementary Materials

The supplementary materials are available online.

## References

[1] Bodor G.S., Porter S., Landt Y., Ladenson J.H. (1992). Development of monoclonal antibodies for an assay of cardiac troponin-I and preliminary results in suspected cases of myocardial infarction. Clin. Chem..

[2] Brien P.J. (2008). Cardiac troponin is the most effective translational safety biomarker for myocardial injury in cardiotoxicity. Toxicology.

[3] Möckel M., Gerhardt W., Heller G., Klefisch F., Danne O., Maske J., Müller C., Störk T., Frei U., Wu A.H. (2001). Validation of NACB and IFCC guidelines for the use of cardiac markers for early diagnosis and risk assessment in patients with acute coronary syndromes. Clin. Chim. Acta.

[4] Cho I., Paek E., Kim Y., Kim J., Paek S. (2009). Chemiluminometric enzyme-linked immunosorbent assays (ELISA)-on-a-chip biosensor based on cross-flow chromatography. Anal. Chim. Acta.

[5] Antonio M., Lupón J., Galán A., Vila J., Zamora E., Urrutia A., Díez C., Coll R., Altimir S., Bayes-Genis A. (2013). Head-to-head comparison of high-sensitivity troponin T and sensitive-contemporary troponin I regarding heart failure risk stratification. Clin. Chim. Acta.

[6] Tuteja S.K., Bhalla V., Deep A., Paul A.K., Suri C.R. (2014). Graphene-gated biochip for the detection of cardiac marker Troponin I. Anal. Chim. Acta.

[7] Jo H., Gu H., Jeon W., Youn H., Her J., Kim S., Lee J., Shin J.H., Ban C. (2015). Electrochemical aptasensor of cardiac troponin I for the early diagnosis of acute myocardial infarction. Anal. Chem..

[8] Li C., Cao D., Qi C., Chen H., Wan Y., Lin Y., Zhang Z., Pang D., Tang H. (2017). One-step separation-free detection of carcinoembryonic antigen in whole serum: Combination of two-photon excitation fluorescence and optical trapping. Biosens. Bioelectron..

[9] Hu G., Sheng W., Zhang Y., Wang J., Wu X., Wang S. (2016). Upconversion Nanoparticles and Monodispersed Magnetic Polystyrene Microsphere Based Fluorescence Immunoassay for the Detection of Sulfaquinoxaline in Animal-Derived Foods. J. Agric. Food Chem..

[10] Wu Q., Li S., Sun Y., Wang J. (2017). Hollow gold nanoparticle-enhanced SPR based sandwich immunoassay for human cardiac troponin I. Microchim. Acta.

[11] Croce A.C., De Simone U., Freitas I., Boncompagni E., Neri D., Cillo U., Bottiroli G. (2010). Human liver autofluorescence: An intrinsic tissue parameter discriminating normal and diseased conditions. Lasers Surg. Med..

[12] Rajendran V.K., Bakthavathsalam P., Ali B.M.J. (2014). Smartphone based bacterial detection using biofunctionalized fluorescent nanoparticles. Microchim. Acta.

[13] Zhou Y., Xia X., Xu Y., Ke W., Yang W., Li Q. (2012). Application of europium (III) chelates-bonded silica nanoparticle in time-resolved immunofluorometric detection assay for human thyroid stimulating hormone. Anal. Chim. Acta.

[14] Lakowicz J.R., Masters B.R. (2008). Principles of fluorescence spectroscopy. J. Biomed. Opt..

[15] Gong X., Tang B., Liu J.J., You X.Y., Gu J., Deng J.Y., Xie W. (2017). Synthesis of adenosine-imprinted microspheres for the recognition of ADP-ribosylated proteins. Biosens. Bioelectron..

[16] Kim D., Bong J., Yoo G., Chang S., Park M., Chang Y.W., Kang M., Jose J., Pyun J. (2016). Microbead-based immunoassay using the outer membrane layer of Escherichia coli combined with autodisplayed Z-domains. Appl. Surf. Sci..

[17] Wang Z., Zong S., Wu L., Zhu D., Cui Y. (2017). SERS-Activated Platforms for Immunoassay: Probes, Encoding Methods, and Applications. Chem. Rev..

[18] Kazemi S.H., Ghodsi E., Abdollahi S., Nadri S. (2016). Porous graphene oxide nanostructure as an excellent scaffold for label-free electrochemical biosensor: Detection of cardiac troponin I. Mater. Sci. Eng. C Mater. Biol. Appl..

[19] Habila M.A., Alothman Z.A., Eltoni A.M., Labis J.P., Khan A., Almarghany A., Elafifi H.E. (2017). One-step carbon coating and polyacrylamide functionalization of Fe_3_O_4_ nanoparticles for enhancing magnetic adsorptive-remediation of heavy metals. Molecules.

[20] Ding L., Zhao M., Fan S., Ma Y., Liang J., Wang X., Song Y., Chen S. (2016). Preparing Co_3_O_4_ urchin-like hollow microspheres self-supporting architecture for improved glucose biosensing performance. Sens. Actuators B Chem..

[21] Liu J., Du B., Zhang P., Haleyurgirisetty M., Zhao J., Ragupathy V., Lee S., Devoe D.L., Hewlett I.K. (2014). Development of a microchip Europium nanoparticle immunoassay for sensitive point-of-care HIV detection. Biosens. Bioelectron..

[22] Cai Y., Chen Y., Hong X., Liu Z., Yuan W. (2013). Porous microsphere and its applications. Int. J. Nanomed..

[23] Heid S., Unterweger H., Tietze R., Friedrich R.P., Weigel B., Cicha I., Eberbeck D., Boccaccini A.R., Alexiou C., Lyer S. (2017). Synthesis and Characterization of Tissue Plasminogen Activator-Functionalized Superparamagnetic Iron Oxide Nanoparticles for Targeted Fibrin Clot Dissolution. Int. J. Mol. Sci..

[24] Song J.S., Chagal L., Winnik M.A. (2006). Monodisperse Micrometer-Size Carboxyl-Functionalized Polystyrene Particles Obtained by Two-Stage Dispersion Polymerization. Macromolecules.

[25] Liu M., Onchaiya S., Tan L., Haghighatbin M.A., Luu T., Owyong T.C., Hushiarian R., Hogan C.F., Smith T.A., Hong Y. (2017). 9-Vinylanthracene based fluorogens: Synthesis, structure-property relationships and applications. Molecules.

[26] Bachmann L., Zezell D.M., Ribeiro A.D.C., Gomes L., Ito A.S. (2006). Fluorescence Spectroscopy of Biological Tissues—A Review. Appl. Spectrosc. Rev..

[27] Bihari P., Vippola M., Schultes S., Praetner M., Khandoga A.G., Reichel C.A., Coester C., Tuomi T., Rehberg M., Krombach F. (2008). Optimized dispersion of nanoparticles for biological in vitro and in vivo studies. Part. Fibre Toxicol..

[28] Sanaeifar N., Rabiee M., Abdolrahim M., Tahriri M., Vashaee D., Tayebi L. (2017). A novel electrochemical biosensor based on Fe_3_O_4_ nanoparticles-polyvinyl alcohol composite for sensitive detection of glucose. Anal. Biochem..

[29] Yin L., Fei L., Cui F., Tang C., Yin C. (2007). Superporous hydrogels containing poly(acrylic acid-acrylamide)/-carboxymethyl chitosan interpenetrating polymer networks. Biomaterials.

[30] Rao P.S., Sathyanarayana D.N. (2002). Inverted emulsion cast electrically conducting polyaniline-polystyrene blends. J. Appl. Polym. Sci..

[31] Liang X., Yue X., Dai Z., Kikuchi J. (2011). Photoresponsive liposomal nanohybrid cerasomes. Chem. Commun..

[32] Mccord J., Nowak R.M., Mccullough P.A., Foreback C., Borzak S., Tokarski G., Tomlanovich M.C., Jacobsen G., Weaver W.D. (2001). Ninety-minute exclusion of acute myocardial infarction by use of quantitative point-of-care testing of myoglobin and troponin I. Circulation.

[33] Song Y., Wang Y., Qi W., Li Y., Xuan J., Wang P., Qin L. (2016). Integrative volumetric bar-chart chip for rapid and quantitative point-of-care detection of myocardial infarction biomarkers. Lab Chip.

[34] Arlati S., Brenna S., Prencipe L., Marocchi A., Casella G.P., Lanzani M., Gandini C. (2000). Myocardial necrosis in ICU patients with acute non-cardiac disease: A prospective study. Intensive Care Med..

[35] Clinical Laboratory Standards Institute (2012). Evaluation of Detection Capability for Clinical Laboratory Measurement Procedures. Approved Guideline.

[36] Bottenus D., Hossan M.R., Ouyang Y., Dong W.J., Dutta P., Ivory C.F. (2011). Preconcentration and detection of the phosphorylated forms of cardiac troponin I in a cascade microchip by cationic isotachophoresis. Lab Chip.

[37] Tsaloglou M.N., Jacobs A., Morgan H. (2014). A fluorogenic heterogeneous immunoassay for cardiac muscle troponin cTnI on a digital microfluidic device. Anal. Bioanal. Chem..

[38] Lee S., Kwon D., Yim C., Jeon S. (2015). Facile detection of Troponin I using dendritic platinum nanoparticles and capillary tube indicators. Anal. Chem..

[39] Liu G., Qi M., Zhang Y., Cao C., Goldys E.M. (2016). Nanocomposites of gold nanoparticles and graphene oxide towards an stable label-free electrochemical immunosensor for detection of cardiac marker troponin-I. Anal. Chim. Acta.

[40] Singal S., Srivastava A.K., Gahtori B., Rajesh (2016). Immunoassay for troponin I using a glassy carbon electrode modified with a hybrid film consisting of graphene and multiwalled carbon nanotubes and decorated with platinum nanoparticles. Microchim. Acta.

[41] Amundson B.E., Apple F.S. (2015). Cardiac troponin assays: A review of quantitative point-of-care devices and their efficacy in the diagnosis of myocardial infarction. Clin. Chem. Lab. Med..

[42] Horak J., Dincer C., Qelibari E., Bakirci H., Urban G. (2015). Polymer-modified microfluidic immunochip for enhanced electrochemical detection of troponin I. Sens. Actuators B Chem..

[43] Kemper D.W., Semjonow V., Theije F.D., Keizer D., Lian V.L., Mair J., Wille B., Christ M., Geier F., Hausfater P. (2016). Analytical evaluation of a new point of care system for measuring cardiac Troponin I. Clin. Biochem..

[44] Guan N., Liu C., Sun D., Xu J. (2009). A facile method to synthesize carboxyl-functionalized magnetic polystyrene nanospheres. Colloids Surf. A.

