# Supplementary material for: Rapid and Sensitive Detection of Cardiac Troponin I for Point-of-Care Tests Based on Red Fluorescent Microspheres
Source: Molecules. 2018 May 7;23(5):1102. doi: 10.3390/molecules23051102 (PMC6099710; doi:10.3390/molecules23051102)
Supplement: Supplementary file 1 [file molecules-23-01102-s001.pdf]

Rapid and sensitive detection of cardiac troponin I for point-of-care tests based on red fluorescent microspheres

Yanxue Cai [1], Keren Kang [2], Qianru Li [1,3], Yu Wang [1] and Xiaowei He [1,*]

[1] School of Food Science and Engineering, South China University of Technology, Guangzhou 510640, China; yanxue.cai@foxmail.com (Y.C.); wyhgn3344@163.com (Y.W.);
[2] National & Local United Engineering Lab of Rapid Diagnostic Test, Guangzhou Wondfo Biotech Co., Ltd., Guangzhou 510663, China; keren.kang@protonmail.com (K.K.)
[3] Fisheries College, Guangdong Ocean University, Zhanjiang 524088, China; qianruli@163.com (Q.L.)
* Correspondence: fexwhe@scut.edu.cn (X.H.); Tel.: +86 020 87556991

1. The Details of Characterization

The morphologies of microspheres were characterized by transmission electron microscope (TEM) (HT7700, Hitachi, Japan). 5 μL of each sample was first dispersed onto a copper grid coated with carbon film, and then was dried at room temperature. The operation was carried out at an acceleration voltage of 200 KV.

The particle size distribution and ζ-potential of blank microspheres and fluorescent nanoparticle were determined using a laser particle size analyzer (Zeta Sizer Nano-S90, Malvern, UK). 3.0 mL samples dispersion was added into polystyrene latex cells, and the mean particle size and ζ-potential were measured at 25 °C with a detector angle of 90°. The PDI was used to evaluate the acceptability of values. The average values from at least five measurements were reported.

Fourier transform infrared spectroscopy (FTIR) was used to determine the changes in functional groups using a FTIR spectrometer (Equinox 55 Bruker Banner Lane, Coventry, Germany). 0.5 mL samples were daubed onto the surface of KBr slice and dried for 2 min with infrared drying lamp. The FTIR spectra were recorded from 4000 to 600 cm^{-1}. For each spectrum, 16 scans at a resolution of 4 cm^{-1} were obtained. All tests were performed at room temperature.

2. Figures

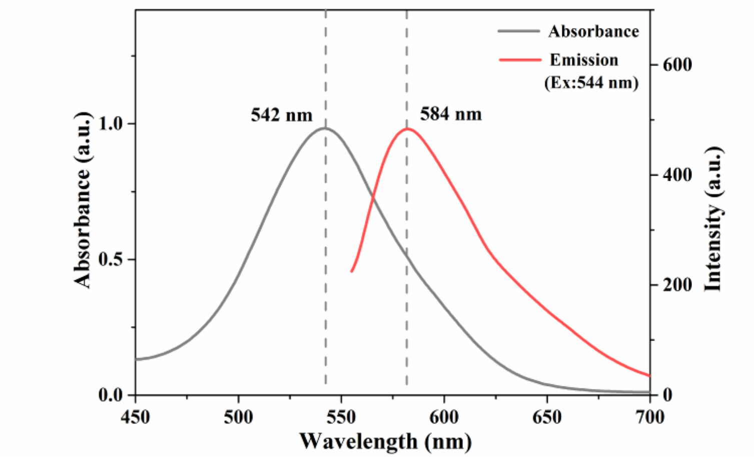

Figure. S1. The absorbance and fluorescence wavelength and intensity of fluorescent microspheres.

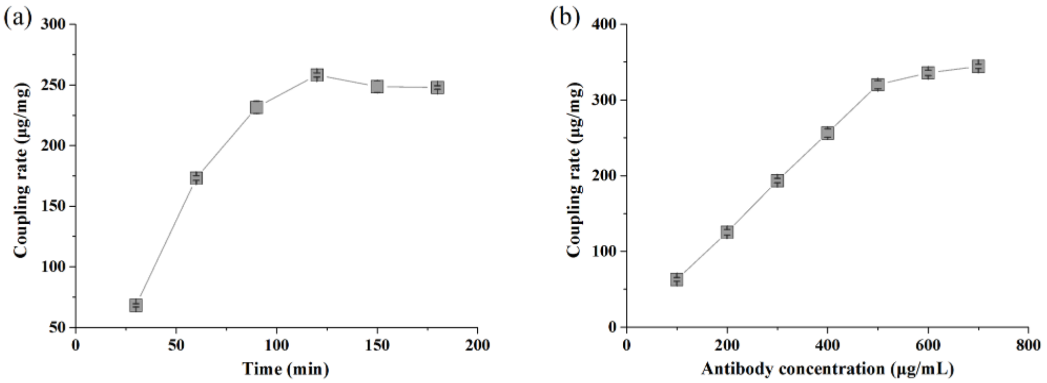

Figure. S2. The reaction time (a) and antibody concentration (b) for the couple between antibody and microspheres.

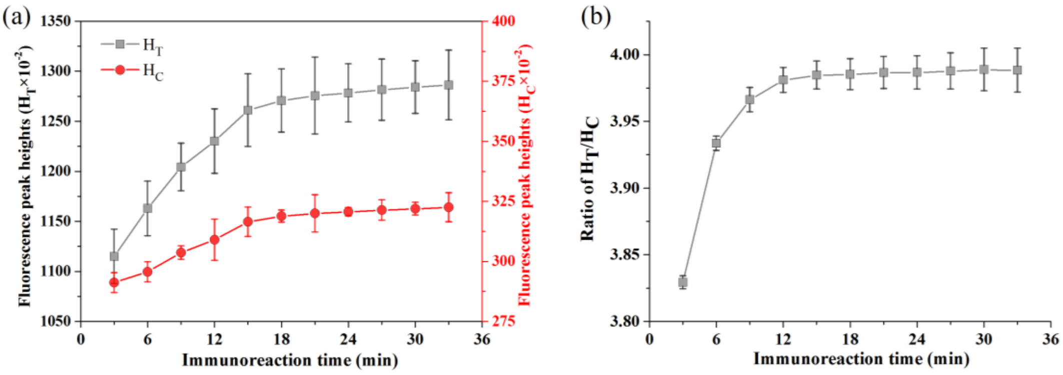

Figure. S3. The immunoreaction time of strip test.

3. Tables

Table. S1. Results of stability analysis by storage and accelerated test.

cTnI (ng/mL)	1.34		2.6		5.7		12		50	
	**Value**	**RD (%)**	**Value**	**RD (%)**	**Value**	**RD (%)**	**Value**	**RD (%)**	**Value**	**RD (%)**
Storage test @ 4 °C										
Day 0	1.47±0.02	8.66	2.71±0.04	4.38	5.75±0.17	0.89	10.99±0.48	-8.38	49.66±2.09	-0.68
Days 90	1.42±0.03	5.82	2.64±0.07	1.54	6.09±0.21	6.86	11.76±0.39	-1.99	45.48±1.93	-9.04
Accelerated test @ 50 °C										
Day 0	1.38±0.02	2.99	2.48±0.11	-4.54	5.96±0.14	4.60	11.88±0.24	-1.01	54.05±0.89	8.09
Days 7	1.22±0.07	-9.18	2.71±0.07	4.04	5.22±0.15	-8.40	11.24±0.31	-6.32	53.75±1.24	7.49
Days 14	1.30±0.04	-2.91	2.83±0.12	9.00	5.23±0.09	-8.33	13.03±0.21	8.55	54.71±2.12	9.42
Days 21	1.26±0.07	-6.04	2.75±0.04	5.73	5.6±0.17	-1.75	12.13±0.35	1.08	53.55±1.93	7.11
Days 28	1.47±0.09	9.40	2.36±0.03	-9.27	5.17±0.16	-9.26	11.09±0.21	-7.62	48.29±2.25	-3.42
